# *Trichoderma* diversity from karst area in Yunnan, Shilin, and four new species

**DOI:** 10.3389/fmicb.2025.1645607

**Published:** 2025-08-08

**Authors:** Xing-Wen Dai, Xian-Kun Zhang, Xiao-Hui Li, Qian-Qian Li, Feng Zhang, Min Qiao, Ming-He Mo, Ying Huang, Ze-Fen Yu

**Affiliations:** ^1^Laboratory for Conservation and Utilization of Bio-Resources, Key Laboratory for Microbial Resources of the Ministry of Education, Yunnan University, Kunming, Yunnan, China; ^2^School of Life Sciences, Yunnan University, Kunming, Yunnan, China

**Keywords:** diversity, karst soils, multi-locus phylogeny, new species, *Trichoderma*

## Abstract

*Trichoderma* spp. are widely distributed across diverse environments and play a significant role in both ecosystem stability and economic applications. In this study, 57 *Trichoderma* strains were isolated from karst desert soil, of which 47 strains were identified as nine known species, while 10 strains were characterized as belonging to four novel species. Phylogenetic analyses, based on the combined sequences of the internal transcribed spacer (ITS), translation elongation factor 1-alpha (*tef1-α*), and RNA polymerase II second largest subunit (*rpb2*) genes, confirmed their distinct taxonomic positions. The results indicate that these four species are distributed across three known clades. Detailed morphological descriptions, cultural characteristics, and illustrations are provided for each new species, and comparisons are made with closely related taxa. The four new species are named *Trichoderma calcicola*, *Trichoderma exigua*, *Trichoderma karsti*, and *Trichoderma xerophilum*. This study documents the diversity of *Trichoderma* in rocky desertification ecosystems that remain agriculturally productive, suggesting their potential ecological adaptation to nutrient-poor, drought-prone, and calcium-rich soils, with implications for future biotechnological and biocontrol applications.

## Introduction

The genus *Trichoderma* (Sordariomycetes, Hypocreales, and Hypocreaceae) exhibits global distribution, is widely found in diverse ecological niches such as soil, plant roots, and decaying wood, and demonstrates remarkable adaptability to various environmental conditions (Cai and Druzhinina, 2021; Cao et al., 2022; Migheli et al., 2009). Moreover, it is recognized for its significant ecological and economic importance. *Trichoderma harzianum* is widely used as a biocontrol agent in the field of agriculture because of its high level of antagonism against diverse phytopathogenic microorganisms (Erazo et al., 2021; Geng et al., 2022; Yan and Khan, 2021; Mitrović et al., 2025). Some *Trichoderma* species have also shown potential in suppressing pathogenic nematodes (Yao et al., 2023). Besides serving biocontrol agents against pathogen, *Trichoderma* species have been shown to promote plant growth (Subramaniam et al., 2022), enhance plant stress tolerance by producing valuable secondary metabolites (Cheng et al., 2012; Mukherjee et al., 2013; Fazeli-Nasab et al., 2022), and facilitate the remediation of soils contaminated with heavy metals (Bandurska et al., 2021; Kidwai et al., 2022). *Trichoderma reesei* and its engineered strains represent significant cellulase producers that are commonly exploited for their carbohydrate-active enzyme content (Sperandio and Filho, 2021). Besides *T. reesei*, several other *Trichoderma* species also produce cellulase, xylanase, and pectinase (Gooruee et al., 2024). However, several *Trichoderma* species pose threats to the cultivation of edible fungi, the production of *Gastrodia elata* BI., and human health (Park et al., 2006; Kim et al., 2012; Sandoval-Denis et al., 2014; Ye et al., 2024).

Rocky desertification (RD), a severe form of karst ecosystem degradation, occurs when progressive soil erosion exposes the underlying bedrock, resulting in substantial agricultural and ecological deterioration. This process advances through multiple soil degradation pathways, including structural collapse, altered soil texture and porosity, reduced water-holding capacity, and nutrient depletion (Huang et al., 2009; Peng et al., 2013; Tang et al., 2013). These processes collectively disrupt ecosystem functioning and generate positive feedback loops that further accelerate RD. The Shilin Karst World Heritage Site in Yunnan Province, renowned for its towering limestone pinnacles, faces increasing threats from RD. Recent studies have demonstrated that the severity of RD drives significant shifts in fungi community composition; as RD intensifies, the abundance of *Penicillium*, *Mortierella*, and *Metarhizium* increases, whereas *Myrothecium*, *Humicola*, *Paramyrothecium*, and *Chaetomium* markedly decline (Yang, 2022). These patterns suggest that microbial indicators may serve as sensitive biomarkers for monitoring RD progression.

The diversity of *Trichoderma* species has been surveyed for different purposes (Mulatu et al., 2022; Cao et al., 2024; Tang et al., 2022). However, the diversity of *Trichoderma* in rocky desertification areas remains unreported. In this study, 57 strains of *Trichoderma* were isolated from the karst rocky desertification soils of Shilin, Yunnan Province. Among these strains, 47 were identified as known species, and 10 were designated as putative new species based on the BLASTn search results of the ITS sequence. To clarify their taxonomic positions, we used an integrative approach based on morphological characteristics and multilocus phylogenetic analyses (ITS, *rpb2*, and *tef1-α*). Furthermore, the analysis revealed significant genetic and morphological differences between the new species and their known counterparts, thereby confirming their status as a novel species. This study not only expands the current understanding of *Trichoderma* diversity in karst desertification ecosystems but also provides a baseline for future research on their ecological functions and potential agricultural applications.

## Materials and methods

### Sample collection and isolation

Soil samples were collected from the rocky desertification region in Shilin County, Kunming City, Yunnan Province (24.6° N, 103.4° E; altitude 1940 m a.s.l.). This area is characterized by exposed bedrock interspersed with gravel, sand, and soil. Despite the degradation, crops such as maize and soybeans are still cultivated, indicating that the region has not reached a fully desertified state. A total of 90 soil samples were collected from three sampling sites, with each located approximately 20 km apart. At each site, 30 samples were collected using a random sampling method, maintaining a minimum spacing of 5 m between sampling points. Samples were taken from a depth of 5–10 cm after the removal of surface plant debris and gravel. All samples were labeled with unique identifiers and detailed collection information. Subsequently, the samples were transferred to the laboratory and stored at 4°C until further analysis.

The soil fungal isolation steps were as follows: 10 g of soil were mixed with 90 mL of sterile water with an appropriate amount of sterile glass beads and then shaken thoroughly at 220 r/min^−1^ for 1 h. After allowing the suspension to stand for 2 min, the supernatant was collected and subjected to serial dilutions (10^−1^ to 10^−4^). Aliquots of 100 μL from the 10^−2^ to 10^−4^ dilutions were plated in triplicate onto Rose Bengal Agar (RBA; Guangdong Huankai Microbial Science and Technology Co., Ltd., China) supplemented with antibiotics (streptomycin, 40 mg/L; ampicillin, 30 mg/L) to suppress bacterial growth. The inoculated plates were incubated in a temperature-controlled chamber at 25°C for 5–7 days and monitored daily for colony growth.

After mycelia growth, well-developed colonies were subcultured onto potato dextrose agar plates (PDA: 200 g potato, 20 g dextrose, 18 g agar, and 1,000 mL distilled water) for further purification and identification. The resulting pure cultures were deposited in the Laboratory for Conservation and Utilization of Bio-Resources, Yunnan University (YMF), Kunming, China.

### Morphology observation

Growth rates were measured on 9-cm-diameter Petri dishes containing three different media: PDA, cornmeal agar (CMA: 20 g cornmeal, 18 g agar, and 1,000 mL distilled water), and synthetic nutrient-poor agar (SNA: 1 g KH_2_PO_4_, 1 g KNO_3_, 0.5 g MgSO_4_, 0.5 g KCl, 0.2 g glucose, 0.2 g sucrose, 18 g agar, and 1,000 mL distilled water), at 25, 30, and 35°C under alternating 12-h light and 12-h dark cycles. After 3 days of incubation, the colony diameter was recorded, and the time required for complete colony coverage was documented. Furthermore, the morphological characters of colonies, such as colony appearance, color, and conidia production, were recorded at the same time. For microscopic morphology, including hyphae, conidiophores, phialides, conidia, and other structures, images were taken using an Olympus BX51 microscope (Tokyo, Japan) connected to a DP controller digital camera. At least 30 datasets were measured for each structure. Colonies were photographed after 7 days, and conidia were photographed after 14 days of production.

### DNA extraction, polymerase chain reaction (PCR) amplification, and sequencing

Genomic DNA was extracted following the method described by Ye et al. (2024). Briefly, 0.5 g of mycelia was transferred into a 2.0-mL microcentrifuge tube, to which steel beads and 700–800 μL of urea extraction buffer [7 mol/L of urea, 50 mmol/L of Tris–HCl, 62.5 mmol/L of NaCl, 10 g/L of sodium dodecyl sulfate (SDS)] were also added, followed by 5 min of disruption at 50 Hz oscillation. The mixture was centrifuged at 12,000 r/min for 5 min, after which the supernatant was transferred to a 1.5-mL centrifuge tube and an equal volume of DNA extraction (phenol/chloroform/isoamyl alcohol, 25:24:1) was added. The mixture was centrifuged at 12,000 r/min for 5 min, and the supernatant was transferred to a new 1.5-mL centrifuge tube, to which an equal volume of isopropanol and 1/10 volume of 3 mol/L of NaAc were added, followed by incubation at −20°C for 20 min. The mixture was then centrifuged at 12,000 r/min for 5 min, and the upper aqueous phase was discarded. The DNA pellets were washed twice with 70% ethanol, dried at 40°C, and then resuspended in 50 μL of sterile water for PCR analysis (Liu et al., 2005).

The ITS, *rpb2*, and *tef1-α* fragments were amplified using three pairs of primers: ITS4 and ITS5 for ITS (White et al., 1990), frpb2-5f and frpb2-7cr for *rpb*2 (Liu et al., 1999), and EF1-728F (Carbone and Kohn, 1999) and TEF1LLErev (Jaklitsch et al., 2005) for *tef1-α*. PCR amplifications were conducted in a 25-μL reaction system containing 12.5 μL of 2 × Master Mix (Accurate Biology), 9.5 μL of double-distilled water, and 1 μL each of forward primer, reverse primer, and DNA template. The PCR reactions were carried out using an Eppendorf Mastercycler (Eppendorf, Hamburg, Germany) following the thermal cycling program described in Table 1. The PCR products were purified using a PCR product purification kit (Biocolor Bioscience and Technology Co., Shanghai, China) and subsequently sequenced in both directions using amplification primers on an ABI 3730 XL DNA sequencer (Applied Biosystems, Foster City, California). The obtained sequences were deposited in the GenBank database at the National Center for Biotechnology Information (NCBI), and the corresponding accession numbers are provided in Table 2.

**Table 1:** PCR primers and thermal cycle programs.

Gene/locus	Primer	Sequence (5–3′)	Thermal cycle programs					
			Prodegenerasation	Denaturation	Annealing	Extension	Final Extension	Cycles
ITS	ITS4	TCCTCCGCTTATTGATATGC (White et al., 1990)	5 min at 94°C	30s at 95°C	30s at 55°C	30s at 72°C	5 min at 72°C	30
	ITS5	GGAAGTAAAAGTCGTAACAAGG (White et al., 1990)						
*rpb2*	fRPB2-5f	GA(T/C)GA(T/C)(A/C)G(A/T)GATCA(T/C)TT(T/C)GG (Liu et al., 1999)	5 min at 94°C	60s at 95°C	60s at 55°C	90s at 72°C	5 min at 72°C	35
	fRPB2-7cr	CCCAT(A/G)GCTTG(T/C)TT(A/G) CCCAT (Liu et al., 1999)						
*tef1-α*	EF1-728F	CATCGAGAAGTTCGAGAAGG (Carbone and Kohn, 1999)	5 min at 94°C	45 s at 95°C	45 s at 55°C	60s at 72°C	5 min at 72°C	35
	TEF1LLErev	AACTTGCAGGCAATGTGG (Jaklitsch et al., 2005)						

**Table 2:** GenBank accession numbers of taxa used in phylogenetic analyses.

Species name	Strain number	GenBank accession number		
		ITS	*rpb2*	*tef1-α*
*T. afroharzianum*	CBS 124620*	FJ442265	FJ442691	FJ463301
*T. afroharzianum*	GJS 04–193	FJ442233	FJ442709	FJ463298
*T. anaharzianum*	YMF 1.00383*	MH113931	MH158995	MH183182
*T. anaharzianum*	YMF 1.00241	MH262584	MH262577	MH236493
*T. aquatica*	YMF 1.04624	MH383057	MK775511	MK775506
*T. aquatica*	YMF 1.04625*	MH383058	MK775512	MK775507
*T. asiaticum*	YMF 1.00352*	MH113930	MH158994	MH183183
*T. asiaticum*	YMF 1.00168	MH262582	MH262575	MH236492
*T. atrobrunneum*	T42	KX632515	KX632572	KX632629
*T. atrobrunneum*	S3	—	KJ665241	KJ665376
*T. azevedoi*	CEN 1422*	MK714902	MK696821	MK696660
*T. azevedoi*	CEN 1423	MK714903	MK696822	MK696661
*T. breve*	HMAS 248844*	KY687927	KY687983	KY688045
*T. breve*	HMAS 2488445	KY687928	KY687984	KY688046
***T. calcicola***	YMF 1.09956*	**PV344624**	**PV366307**	**PV346763**
***T. calcicola***	**YMF 1.09957**	**PV344625**	**PV366308**	**PV346764**
*T. camerunense*	GJS 99–230	NR137300	—	AF348107
*T. camerunense*	GJS 99–231	AY027783	—	AF348108
*T. endophyticum*	DIS 220 K	FJ442270	FJ442765	FJ463328
*T. endophyticum*	DIS 220 J	FJ442254	FJ442690	FJ463330
***T. exigua***	YMF 1.10219*	**PV702217**	**PV711372**	**PV711374**
***T. exigua***	**YMF 1.10220**	**PV702218**	**PV711373**	**PV711375**
*T. gamsii*	S488	—	KJ665270	JN715613
*T. gamsii*	G. J. S. 04–09	DQ315459	JN133561	DQ307541
*T. ghanense*	18ASMA008	MT520628	—	MT671928
*T. ghanense*	18ASMA007	MT520627	—	MT671927
*T. guizhouense*	HGUP 0038	JN191311	JQ901400	JN215484
*T. hailarense*	WT 17901*	MH287485	MH287506	MH287505
*T. harzianum*	CBS 226.95*	AJ222720	AF545549	AF348101
*T. harzianum*	GJS 05–107	FJ442679	FJ442708	FJ463329
*T. hispanicum*	S453T	JN715595	JN715600	JN715659
***T. karsti***	YMF 1.09950*	**PV344618**	**PV366301**	**PV346757**
***T. karsti***	**YMF 1.09951**	**PV344619**	**PV366302**	**PV346758**
*T. lentiforme*	DIS 218E	FJ442220	FJ442793	FJ463310
*T. lentiforme*	DIS 173F	FJ442253	FJ442787	FJ463347
*T. lentinulae*	CGMCC 3.19848	MN594470	MN605868	MN605879
*T. lentinulae*	HMAS 248256*	MN594469	MN605867	MN605878
*T. lixii*	CBS 110080	NR_131264	KJ665290	FJ716622
*T. longibrachiatum*	C. P. K. 1707	—	JN182315	EU401610
*T. longibrachiatum*	S 328	—	JQ685883	JQ685867
*T. longiphialidicum*	TC 675	—	MF095872	MF095880
*T. longiphialidicum*	TC 668	—	MF095871	MF095879
*T. macrochlamydospora*	JZBQT5Z1*	ON653399	ON649955	ON649902
*T. miyunense*	JZBQF7*	—	ON649969	ON649916
*T. miyunense*	JZBQF9	—	ON649970	ON649917
*T. neokoningii*	CBS 120070	MH863076	KJ665318	KJ665620
*T. notatum*	JZBQT1Z5*	—	OP832381	OP832396
*T. parareesei*	CBS 125925*	—	HM182963	GQ354353
*T. parareesei*	C. P. K. 634	—	HM182968	GQ354351
*T. pholiotae*	JZBQH12*	—	ON649972	ON649919
*T. pingquanense*	JZBQT7Z10*	ON653401	ON649961	ON649908
*T. propepolypori*	YMF 1.06224*	MN977789	MT052181	MT070158
*T. propepolypori*	YMF 1.06199	MN977790	MT052182	MT070157
*T. pseudoasiaticum*	YMF 1.06200*	MN977792	MT052183	MT070155
*T. pseudopyramidale*	COAD 2426*	—	MK044224	MK044131
*T. pseudopyramidale*	COAD 2427	—	MK044229	MK044136
*T. pyramidale*	CBS 135574	—	KJ665334	KJ665699
*T. reesei*	G. J. S. 00–89	—	JN175548	JN175599
*T. reesei*	G. J. S. 97–38	AJ004962	JN175552	JN175603
*T. rifaii*	CBS 130746*	FJ442663	—	FJ463324
*T. rifaii*	DIS 337F	FJ442621	FJ442720	FJ463321
*T. rugosum*	HMAS254536	—	MH612372	MH612378
*T. rugosum*	HMAS254548	—	MH612373	MH612379
*T. rugulosum*	SFC20180301-001*	MH050353	MH025986	MH025984
*T. rugulosum*	SFC20180301-002	—	MH025987	MH025985
*T. samuelsii*	S42	JN715593	JN715598	JN715652
*T. sempervirentis*	CBS 133498*	—	KC285755	KC285632
*T. sempervirentis*	S601	—	KC285756	KC285633
*T. simile*	YMF 1.06201*	MN977793	MT052184	MT070154
*T. simile*	YMF 1.06202	MN977794	MT052185	MT070153
*T. subvermifimicola*	JZBQT4Z1*	ON653398	ON649952	ON649899
*T. thermophilum*	HMAS252912	—	KX066261	KX066249
*T. tongzhouense*	JZBQT1Z1*	ON653394	ON649945	ON649892
*T. vermifimicola*	CGMCC 3.19850	MN594472	MN605870	MN605881
*T. vermifimicola*	HMAS 248255*	MN594473	MN605871	MN605882
***T. xerophilum***	**YMF 1.09952**	**PV344620**	**PV366303**	**PV346759**
***T. xerophilum***	YMF 1.09953*	**PV344621**	**PV366304**	**PV346760**
***T. xerophilum***	**YMF 1.09958**	**PV344622**	**PV366305**	**PV346761**
***T. xerophilum***	**YMF 1.09959**	**PV344623**	**PV366306**	**PV346762**
*Protocrea farinosa*	CBS 121551	MH863119	EU703935	EU703889
*Protocrea pallida*	CBS 299.78	MH861137	EU703948	EU703900

^1^Novel species introduced in this study are indicated in bold. ^2^The type and ex-type strains are indicated with * after the strain number. ^3^″—” indicates sequence unknown.

### Sequence alignment and phylogenetic analyses

Preliminary BLASTn searches were conducted using the ITS, *rpb2*, and *tef1-α* sequences of the newly isolated strains against the NCBI database to identify closely related species. Both the reference sequences and the newly generated sequences in this study are listed in Table 2. Phylogenetic reconstruction was performed based on the concatenated sequences of the ITS, *rpb*2, and *tef1-α* loci. Sequence alignment was conducted using Clustal X 1.83 (Thompson et al., 1997) with default parameters, followed by trimming to appropriate lengths using MEGA11 (Tamura et al., 2021). Sequence assembly and alignment were carried out in BioEdit version 7.0 (Hall, 1999), with manual concatenation of the aligned sequences from the three loci. Missing nucleotide positions were filled with question marks “?” to facilitate subsequent analyses and to optimize the quality of sequence assembly. A sequence matrix (FASTA file) containing three gene loci was generated using BioEdit version 7.0, with a total of 3,024 characters (669 from ITS, 1,041 from *rpb*2, and 1,314 from *tef1-α*). The alignment data used in the phylogenetic analyses were deposited in TreeBASE.

Phylogenetic reconstruction of the newly identified species was conducted through both maximum likelihood (ML) and Bayesian inference (BI) approaches. For the ML analysis, the concatenated sequence matrix in FASTA format, assembled using BioEdit version 7.0 (Hall, 1999), was analyzed in IQ-TREE software (Nguyen et al., 2015). The optimal nucleotide substitution model was selected through ModelFinder, executed with the command iqtree -s example.fas -m MF -nt AUTO, which identified the TNe + I + G4 model as the best-fit evolutionary model based on the Bayesian Information Criterion (BIC). Bootstrap support values were estimated from 1,000 replicates following the outgroup designation. Bayesian trees were constructed using MrBayes v3.1.2 (Huelsenbeck and Ronquist, 2001), with the best model chosen through MrModeltest 2.3. The Markov chain Monte Carlo (MCMC) analysis was initiated with four parallel chains (one cold and three heated) per run, which proceeded for five million generations with sampling intervals of 500 generations until the average standard deviation of split frequencies fell below 0.01. The initial 25% of sampled generations were discarded as burn-in, with the remaining samples utilized to compute posterior probabilities for Bayesian phylogenetic reconstruction. Phylogenetic trees were visualized using FigTree version 1.4, with the nodal support values indicated by both maximum likelihood bootstrap proportions (MLBPs≥75%) and Bayesian posterior probabilities (BIPPs≥0.85).

## Results

### Diversity analysis

A total of 57 strains of *Trichoderma* were isolated and purified from rocky desertification soils based on the initial colony morphology. Among these strains, 47 were identified as known species, and 10 were designated as putative new species based on the BLASTn search results of the ITS sequence.

Phylogenetic analyses inferred from the ITS sequence were conducted to identify known species. The detailed species and their isolation frequencies are provided in Table 3. The highest isolation frequency was observed in *T. harzianum*, reaching 26.31%. The isolation frequencies of the remaining species were as follows: 12.28% each for *Trichoderma koningiopsis* and *Trichoderma sulphureum*, 8.77% for *Trichoderma gamsii*, 7.02% for *T. hamatum*, 5.26% for *T. virens*, and 3.51% each for *T. atroviride*, *T. cerinum*, and *T. spirale*.

**Table 3:** Isolated known species and frequency.

Species	GenBank	Number	Frequency
*T. atroviride* P. Karst	451289	2	3.51%
*T. cerinum* Bissett, Kubicek & Szakács	488349	2	3.51%
*T. gamsii* Samuels & Druzhin	501050	5	8.77%
*T. hamatum* (Bonord.) Bainie*r*	165799	4	7.02%
*T. harzianum* Rifai	340299	15	26.31%
*T. koningiopsis* Samuels, Carm. Suárez & Evans	487454	7	12.28%
*T. spirale* Bissett	359087	2	3.51%
*T. sulphureum* (Schwein.) Jaklitsch and Voglmayr	807456	7	12.28%
*T. virens* (Mill., Giddens & Foster) Arx	128198	3	5.26%

### Phylogenetic analyses

**Figure 1:**
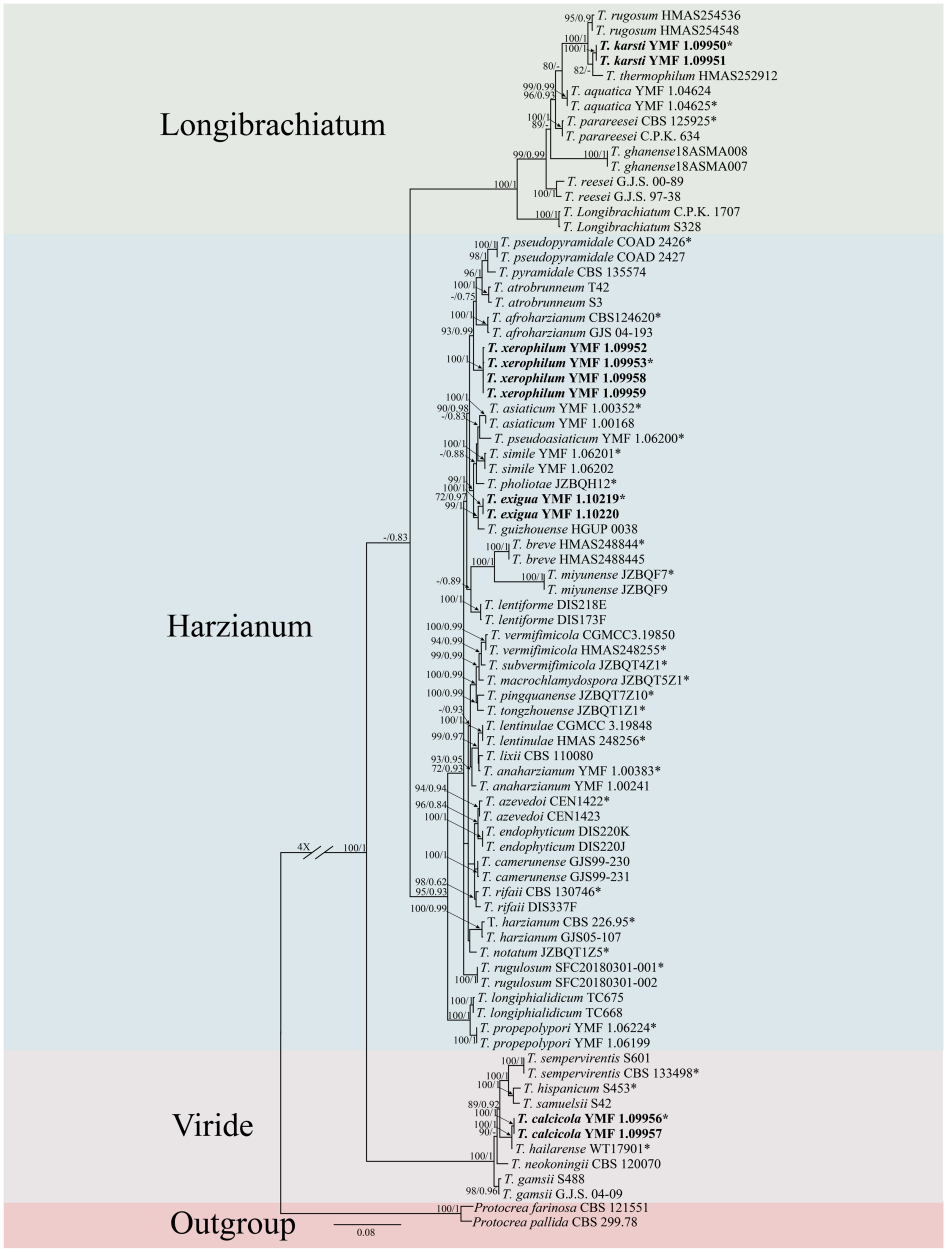
Phylogenetic tree of *Trichoderma* species based on the combined ITS, *rpb2*, and *tef1-α* gene sequences constructed using the maximum likelihood (ML) analysis and Bayesian inference (BI) analysis. The numbers above branches represent maximum-likelihood bootstrap percentages (left) and Bayesian posterior probabilities (right). ML bootstrap support (70) and Bayesian posterior probabilities (0.75) are shown on the respective branches. *Protocrea farinose* CBS 121551 and *P. pallida* CBS 299.78 were used as outgroups. Bold font indicates newly described species.

A concatenated dataset comprising ITS, *rpb2* and *tef1-α* sequences (total length: 3,024 characters) was analyzed to determine the phylogenetic placement of the novel species. Phylogenetic trees were reconstructed through both ML and BI methods and exhibited consistent topological structures (Figure 1). Based on combined morphological characteristics and phylogenetic evidence, 10 isolates were identified as 4 new *Trichoderma* species, which are distributed across three different clades. The four new species were proposed as *T. calcicola*, *T. exigua, T. karsti*, and *T. xerophilum*, and each was supported by robust phylogenetic evidence and distinct morphological characteristics.

Two isolates were assigned to the Longibrachiatum clade, forming a new subclade corresponding to a novel species, designated as *T. karsti* (MLBP/BIPP = 100/1.00). In the Viride clade, two isolates formed a new subclade, defined as a novel species, designated as *T. calcicola* (MLBP/BIPP = 100/1.00). In the Harzianum clade, six isolates formed two new subclades, which were identified as novel species and named *T. xerophilum* (MLBP/BIPP = 100/1.00) and *T. exigua* (MLBP/BIPP = 100/1.00).

### Taxonomy

**Figure 2:**
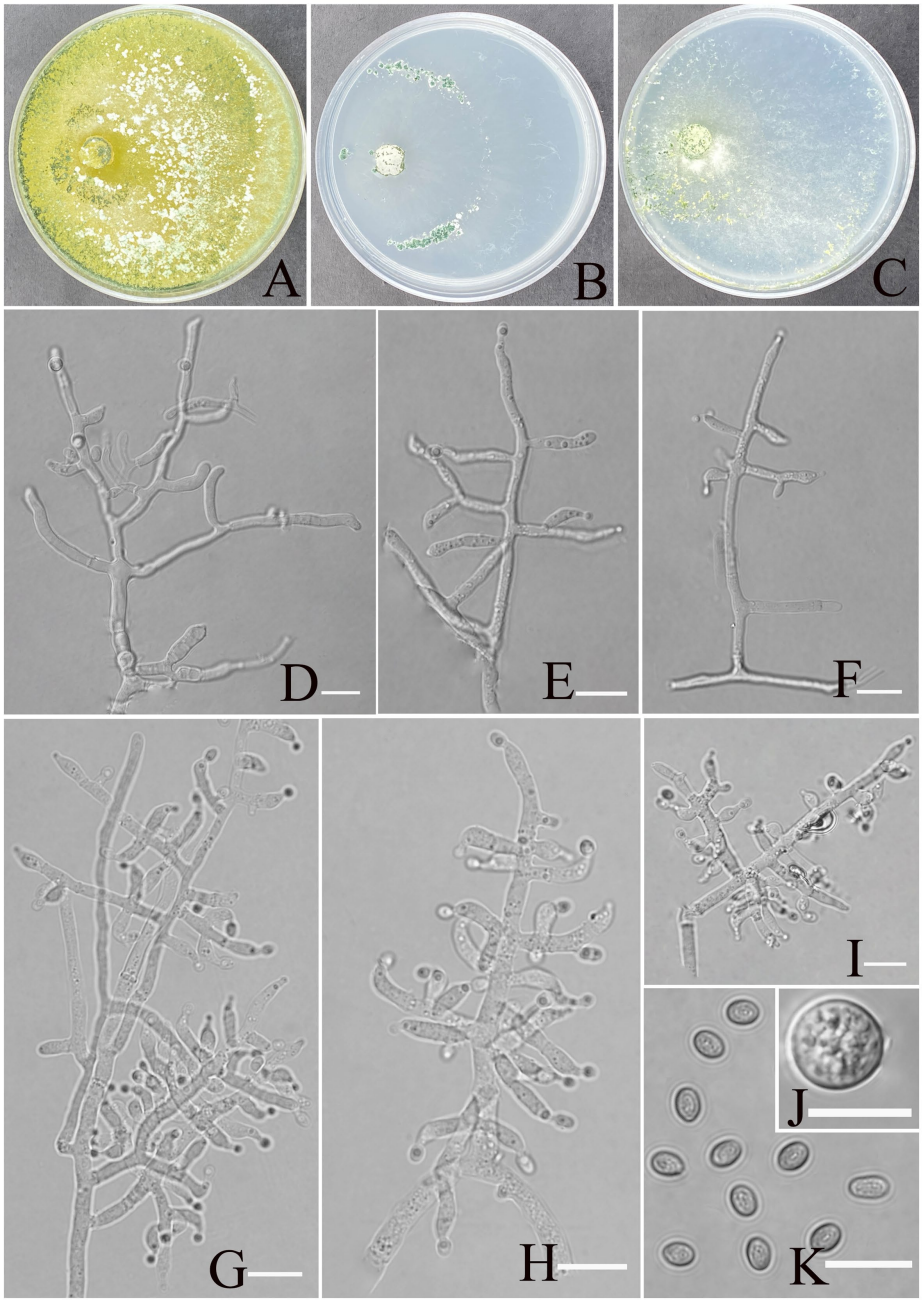
Morphology of *Trichoderma karsti* (YMF1.09950). **(A–C)** Cultures on PDA plates, 7d; CMA plates, 7d; SNA plates, 7d; 25°C; **(D–I)** conidiophores and phialides; **(J)** chlamydospores; and **(K)** conidia. Scale bars: 10 μm **(D–K)**.

***Trichoderma karsti* Z. F. Yu & X. W. Dai, sp. nov.** Figure 2.

**MycoBank No:** 860053.

**Etymology:** Latin, *karsti*, refers to the holotype being isolated from karst soil.

**Description:** Sexual morph: Unknown. **Asexual morph:** Conidiophores consisting of a recognizable main axis with branches arranged either in pairs or singly, arising at an angle slightly less than 90° with respect to the main axis. The distance between two neighboring branches ranges from 3.5 to 13.2 μm. Phialides are commonly singly, opposite, ampulliform or narrowly vase-shaped, and the tip is long and curved, oriented an indefinite direction. They measure (6.2–)7.5–9.5(−11.3) × 2.3–4.1 μm, with a length-to-width (l/w) ratio of 1.9–4.6(−5.1), and, at base, they are 1.6–2.4(−3.5) μm wide and widest around the middle. Conidia are oval, elliptic, pale yellow-green, and smooth-walled, measuring 3.8–5.2 × 2.8–3.2 μm, with an l/w ratio of 1.1–1.4. Chlamydospores were observed growing at the tip of hyphae, round, measuring 8.2–10.8 × 7.1–9.7 μm, with a l/w ratio of 1.0–1.1.

**Culture characteristics:** optimum temperature for growth 30°C.

After 72 h, the colony radius on PDA was 64 mm at 25°C, 72 mm at 30°C, and 59 mm at 35°C, covering the plate after 3 days at 30°C. The colony is white, circular, and turns green after 3 days. Aerial hyphae are abundant, forming a dense mat. Pure yellow pigments are noted. A slight odor was noted.

Colony radius on CMA after 72 h: 12 mm at 25°C, 29 mm at 30°C, and 15 mm at 35°C. The colony lucency is circular, darkening to deep green as the incubation time extended. There was no diffusing pigment noted, and the odor was indistinct.

Colony radius on SNA after 72 h: 13 mm at 25°C, 34 mm at 30°C, and 30 mm at 35°C. The colony is white and turns green after 5 days. Sulphur yellow pigment was noted, and a slight odor was noted. Chlamydospores noted in all media.

**Materials examined:** China, Yunnan Province, Shilin Country, from soil of rocky desertification, August 2024, Z. F. Yu, (holotype YMF 1.09950). lbid. (cultures: YMF 1.09951).

**Notes:** From a systematic perspective, *T. karsti* is closely related to *T. thermophilum* and associated with *T. rugosum*. *T. thermophilum* and *T. rugosum*, which exhibit a sexual morph, *T. karsti* has only been observed in its asexual state (Qin and Zhuang, 2016; Zhang and Zhuang, 2018). The phialides of *T. thermophilum* and *T. rugosum* are relatively regular in morphology, while those of *T. karsti* are more curved and asymmetrical. In addition, the conidia of *T. karsti* are larger than those of *T. thermophilum* (3.8–5.2 × 2.8–3.2 vs. 2.7–6 × 2.3–3) and *T. rugosum* (3.8–5.2 × 2.8–3.2 vs. 3–4 × 2.2–3). The colonies of all three species are yellow on PDA and CMA, while they appear transparent or translucent on SNA. *T. karsti* has a mild odor, whereas no distinct odor was detected in the other two species.

**Figure 3:**
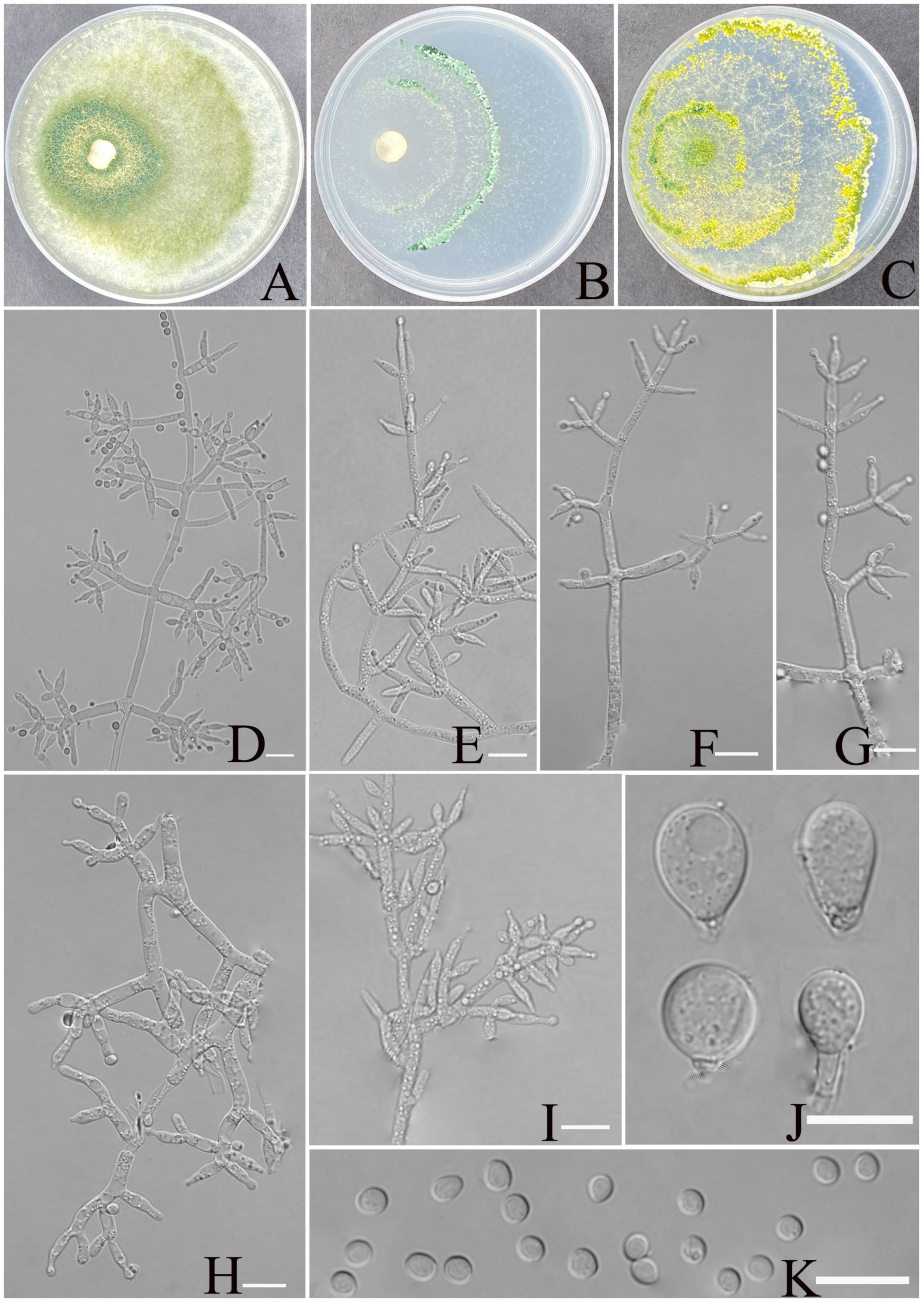
Morphology of *Trichoderma xerophilum* (YMF1.09953). **(A–C)** Cultures on PDA plates, 7d; CMA plates, 7d; SNA plates, 7d; 25°C; **(D–I)** conidiophores and phialides; **(J)** chlamydospores; and **(K)** conidia. Scale bars: 10 μm **(D–K)**.

***Trichoderma xerophilum* Z. F. Yu & X. W. Dai, sp. nov.** Figure 3.

**MycoBank NO:** 860054.

**Etymology:** Latin, *xerophilum*, refers to the arid karst soil.

**Description:** sexual morph: unknown. **Asexual morph:** conidiophores comprising a recognizable main axis, primary branches that are mostly paired, occasionally 3 verticillate or solitary, and arise at an angle of approximately 90° from the main axis. Each branch terminates in a paired and a whorl of 3 together with a terminal phialide. Phialides ampulliform to narrowly vase-shaped, straight or slightly curved, mostly paired or whorls of 3 on terminal branches of the conidiophore, occasionally solitary, (5.5–)6.0–8.7(−11.5) × 2.1–3.2 μm, l/w ratio 2.3–4.9, 1.4–3.2 μm wide at base, widest around the middle. Conidia are oval, elliptic to subspheroidal, green, smooth, (3.1–)3.3–3.9(−4.2) × (2.5–)2.7–3.4 (−3.6) μm, with a l/w ratio of 1.0–1.2. The chlamydospores observed at the tips of hyphae exhibited two distinct morphological types: elliptical, measuring 10.0–12.9 × 7.8–9.2 μm with a length-to-width ratio of 1.2–1.6, and subglobose, measuring 8.5–11.2 × 6.6–9.4 μm with a length-to-width ratio of 1.0–1.2.

**Culture characteristics:** Optimum temperature for growth is 30°C.

Colony radius on PDA after 72 h: 55 mm at 25°C, 63 mm at 30°C, and 40 mm at 35°C, covering the plate after 3 days at 30°C. The colony is translucent, circular, and radial, with a white to pale grayish green. Aerial hyphae are abundant, forming a dense mat. Pure yellow pigments noted, slight odor noted.

Colony radius on CMA after 72 h: 25 mm at 25°C, 32 mm at 30°C, and 29 mm at 35°C. The colony lucency is circular, 1–2 zonate, darkening to green as incubation time extended. No diffusing pigment was noted, and odor was indistinct.

Colony radius on SNA after 72 h: 44 mm at 25°C, 50 mm at 30°C, and 30 mm at 35°C. The colony lucency is circular, three or more zonate, and the color changes to yellowish green after 3 days. Pure yellow pigments were noted, and a slight odor was noted. Chlamydospores were noted in all media.

**Materials examined:** China, Yunnan Province, Shilin Country, from soil of rocky desertification, August 2024, Z. F. Yu, (holotype YMF 1.09953). lbid. (cultures: YMF 1.09952, YMF 1.09958, YMF 1.09959).

**Notes:** Based on phylogenetic analyses, the four strains of *T. xerophilum* formed a single clade, sistering to the clade formed by *T. afroharzianum*, *T. atrobrunneum*, *T. pyramidale,* and *T. pseudopyramidale*. The phialides of *T. afroharzianum* (5.2–10.2 × 2.0–3.5 μm) and *T. atrobrunneum* (5.5–8.0 × 2.2–3.7 μm) are lageniform to ampulliform, whereas *T. xerophilum* exhibits longer phialides (6.0–8.7 × 2.4–3.2 μm) with a more pronounced length-to-width ratio, making it distinctive within the group. *T. atrobrunneum* does not mention the formation of chlamydospores, while *T. afroharzianum* rarely produces them (Chaverri et al., 2015). In contrast, *T. xerophilum* forms chlamydospores at the tips of hyphae, exhibiting two distinct morphological types. Furthermore, the phialides of *T. pyramidale* exhibit greater morphological diversity, ranging from lageniform to ampulliform and occasionally inequilateral or sigmoid, compared to the more uniform phialides of *T. xerophilum* (5.5–11.5(−17.5) × 2.8–3.7(−4.5) vs. 6.0–8.7(−11.5) × 2.1–3.2), thereby reflecting the morphological differences between the two species (Chaverri et al., 2015). Similarly, the phialides of *T*. *pseudopyramidale* (5.3–8.6(−9.1) × 2.2–2.9(−3.2) μm) are predominantly ampulliform to lageniform and usually formed in whorls, showing a somewhat narrower width than those of *T. xerophilum* (6.0–8.7(−11.5) × 2.1–3.2 μm) (del Carmen et al., 2021). These morphological characteristics distinctly set *T. xerophilum* apart from other closely related species.

**Figure 4:**
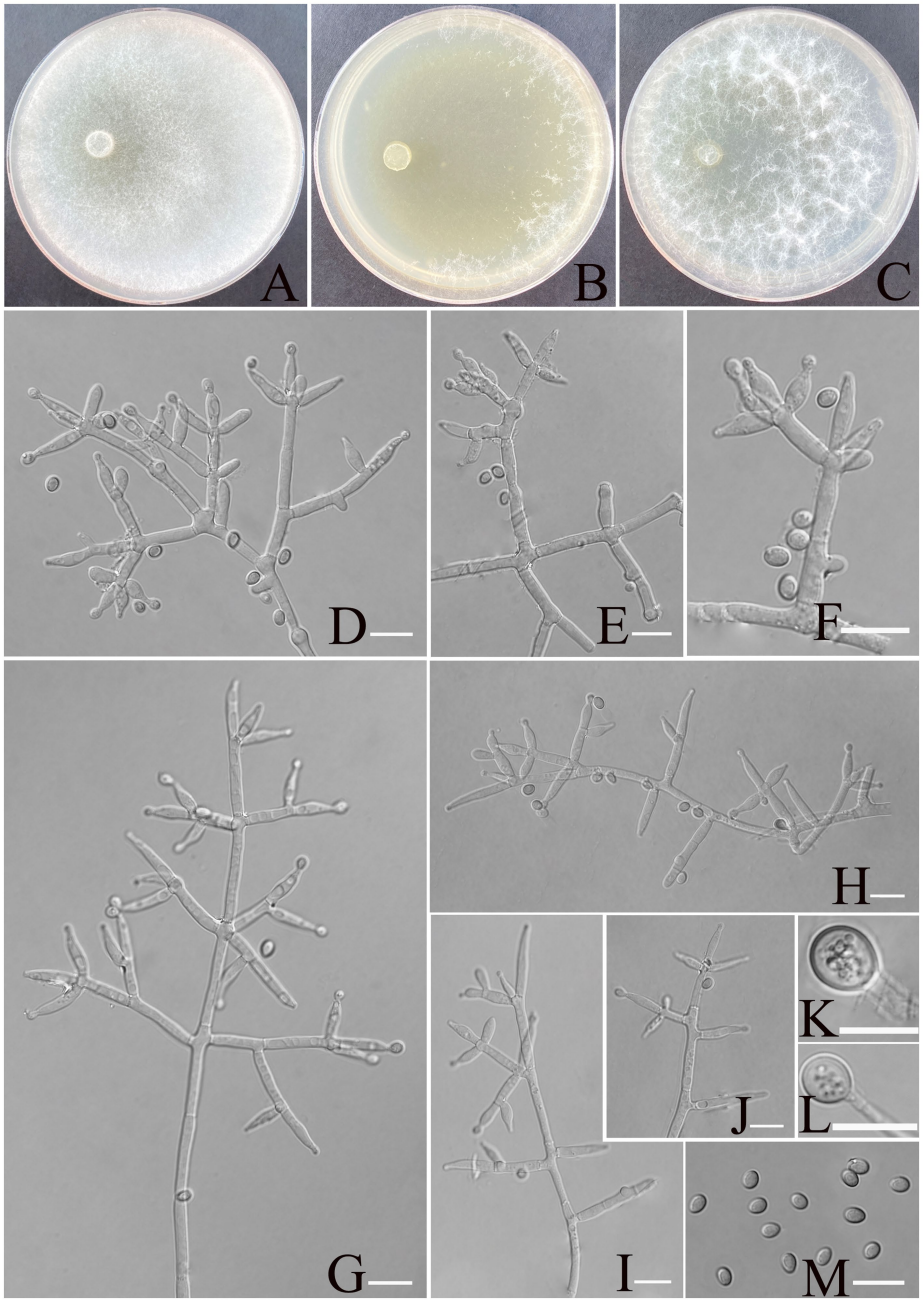
Morphology of *Trichoderma calcicola* (YMF1.09956). **(A–C)** Cultures on PDA plates, 7d; CMA plates, 7d; SNA plates, 7d; 25°C; **(D–J)** conidiophores and phialides; **(K,L)** chlamydospores; and **(M)** conidia. Scale bars: 10 μm **(D–K)**.

***Trichoderma calcicola* Z. F. Yu & X. W. Dai, sp. nov.** Figure 4.

**MycoBank NO:** 860055.

**Etymology:** Latin, *calcicola*, referring to the limestone-rich karst soil from which the strain was isolated.

**Description:** Sexual morph: Unknown. Asexual morph: Conidiophores and branches form a pyramidal structure, the distance between two neighboring branches (6.7–)8.0–25.0(−30.0) μm. Branches paired asymmetrically or solitary, occasionally in a whorl of 3 at an angle less than or near 90° concerning the main axis, branches terminating in a single, paired, or a whorl of three phialides. Phialides are spindle-shaped and lageniform, (6.4–)7.5–11.4(−12.0) × (2.6–)2.9–3.8(−4.0) μm, l/w ratio 1.4–3.5. Conidia thin-walled, ellipsoidal, rarely globose, green, smooth, (3.5–)3.8–4.5(−4.8) × (2.6–)2.8–3.2(−3.5) μm, l/w ratio 1.2–1.4. Chlamydospores were noted at the tip of hyphae, round, and measure 7.1–10.23 × 6.2–8.4 μm, with a l/w ratio of 1.1–1.2.

**Culture characteristics:** Optimum temperature for growth is 25°C.

Colony radius on PDA after 72 h: 55 mm at 25°C, 50 mm at 30°C, and 24 mm at 35°C, covering the plate after 3 days at 25°C. The colonies are white, circular, and fuzzy; aerial hyphae are abundant. No diffusing pigment noted, slight odor noted.

Colony radius on CMA after 72 h: 35 mm at 25°C, 32 mm at 30°C, and 24 mm at 35°C. The colony lucency is circular, the central air mycelia of the colony exiguity, and the margin dense. No diffusing pigment noted, slight odor noted.

Colony radius on SNA after 72 h: 40 mm at 25°C, 33 mm at 30°C, and 30 mm at 35°C. The colonies are white, circular, and fuzzy, aerial hyphae hairy to floccose, dense. Slight odor noted. Chlamydospores were observed in all media.

**Materials examined:** China, Yunnan Province, Shilin Country, from soil of rocky desertification, August 2024, Z. F. Yu, (holotype YMF 1.09956). lbid. (cultures: YMF 1.09957).

**Notes:** *T. calcicola* and *T. hailarense* are phylogenetically related but exhibit distinct differences in morphological and culture characteristics (Zhang et al., 2022). Regarding phialides, *T. hailarense* features longer lageniform phialides (8.0–15.5 μm × 2.5–3.6 μm), while *T*. *calcicola* possesses spindle- to lageniform-shaped phialides (6.4–12.0 μm × 2.6–4.0 μm). For conidia, *T. hailarense* yields delicately roughened, obovoid conidia (4.2–4.9 μm × 3.4–4.1 μm), whereas *T*. *calcicola* produces smooth, ellipsoidal conidia (3.5–4.8 μm × 2.6–3.5 μm). *T. hailarense* exhibits faster growth at 30°C, whereas *T. calcicola* shows better adaptation to growth conditions at 25°C.

**Figure 5:**
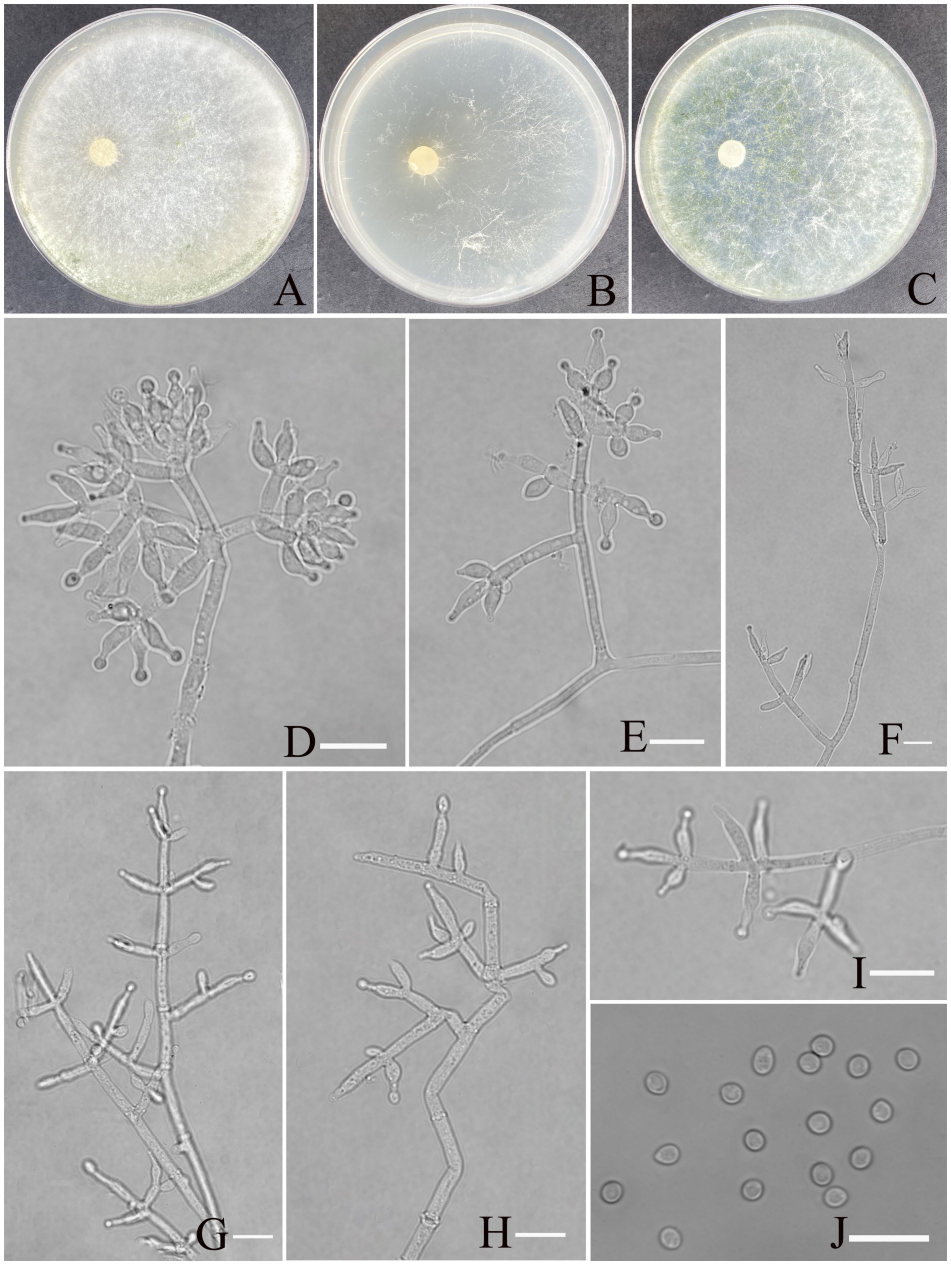
Morphology of *Trichoderma exigua* (YMF 1.10219). **(A–C)** Cultures on PDA plates, 7d; CMA plates, 7d; SNA plates, 7d; 25°C; **(D–I)** conidiophores and phialides; **(J)** conidia. Scale bars: 10 μm **(D–J)**.

***Trichoderma exigua* Z. F. Yu & X. W. Dai, sp. nov.** Figure 5.

**MycoBank NO:** 860056.

**Etymology:** Latin, *exigua*, refer exiguous conidiation.

**Description:** Sexual morph: Unknown. Asexual morph: Conidiophores more or less symmetrical, main axis recognizable, branches arising at an angle of less than 90° concerning the main axis. Most branches are paired or form a whorl of 3, occasionally solitary. Phialides concentrated on the apex of conidiophores, arranged in 2–5 whorls, less solitary, ampulliform or narrowly vase-shaped, straight or curved, with an indefinite direction. (5.5–)6.5–9.7(−12.0) × (2.0–) 2.6–3.5(−4.2) μm, l/w ratio1.6–3.5. Conidia oval, elliptic, green, smooth, (2.7–)2.9–3.5(−4.1) × (2.4–)2.5–2.8(−3.0) μm, l/w ratio 1.1–1.3. Chlamydospores not found.

**Culture characteristics:** Optimum temperature for growth is 30°C.

Colony radius on PDA after 72 h: 51 mm at 25°C, 60 mm at 30°C, and 42 mm at 35°C, covering the plate after 3 days at 30°C. The colony is white, circular, and turns primrose after 3 days. Aerial hyphae are abundant, forming a dense mat. No diffusing pigment noted, slight odor noted.

Colony radius on CMA after 72 h: 30 mm at 25°C, 35 mm at 30°C, and 27 mm at 35°C. The colony is lucency, with the air mycelium having more edges and less center; no diffusing pigment was noted, and odor was indistinct.

Colony radius on SNA after 72 h: 40 mm at 25°C, 51 mm at 30°C, and 33 mm at 35°C. The colony is white, circular. Three days later, the center of the colony turns yellow-green. No diffusing pigment was noted, and odor was indistinct.

**Materials examined:** China, Yunnan Province, Shilin Country, from soil of rocky desertification, August 2024, Z. F. Yu, (holotype YMF 1.10219). lbid. (cultures: YMF 1.10220).

**Notes:** *T. exigua* and *T. guizhouense* are phylogenetically related but exhibit distinct differences in morphological and culture characteristics (Li et al., 2013). However, *T. exigua* possesses distinctly longer lageniform phialides than the ampulliform to lageniform phialides of *T. guizhouense* (6.5–9.7 × 2.6–3.5 vs. 4.5–10 × 2–3); the phialides of the former are organized in 2–5 whorls, while those of the latter are often in a whorl of 3. Moreover, conidia of *T. exigua* are smooth, oval to elliptic, and larger (2.4–3.0 vs. 2–3), while conidia of *T. guizhouense* are globose.

Finally, regarding culture characteristics, *T. guizhouense* exhibits rapid growth, with a colony radius of 57–58 mm on PDA at 25°C after 72 h, whereas *T. exigua* shows slower growth. Both species lack diffusing pigments, although *T. guizhouense* may produce a brown diffusing pigment in some strains, and both species emit a slight odor.

## Discussion

Current taxonomic resolution within the *Trichoderma* genus has been achieved through integrative analyses incorporating phylogenetic, morphological, ecological, and biogeographical data. Notably, two genetic loci, *rpb2* and *tef1-α*, have been established as the standard molecular markers for the identification of novel *Trichoderma* species (Cao et al., 2024). These molecular markers, along with comprehensive morphological examination, have significantly enhanced the precision of species delimitation within this genus. This study used a comprehensive analysis of multi-gene sequences (ITS, *rpb2* and *tef1-α*) along with morphological characteristics to systematically elucidate the phylogenetic relationships among the species. Based on the multi-gene phylogenetic tree, the four new species were classified into three distinct clades: Longibrachiatum, Viride, and the Harzianum clades. Furthermore, all clades exhibited high maximum likelihood bootstrap proportions and Bayesian posterior probabilities, providing strong support for their phylogenetic classification.

The newly described species *T. calcicola* belong to the Viride clade, one of the most species-rich and widely distributed clades within the genus *Trichoderma*. The Viride clade, initially referred to as the “section *Trichoderma*,” is represented by the type species *T. viride* Pers (Bissett, 1991). Building upon the study of Samuels et al. (2006), Jaklitsch et al. (2013) further analyzed the complex group; subsequently, Jaklitsch and Voglmayr (2015) formally renamed the clade the Viride clade through the construction of an updated phylogenetic tree. Species in this clade primarily exhibit verticillate or pachybasium-like conidiophores, with phialides arranged in whorls or pairs and producing green conidia, yet they display significant diversity in colony morphology, growth rates, and conidial shape and size (Qin and Zhuang, 2016). Members of this clade demonstrate remarkable ecological versatility, having been isolated from diverse substrates such as decaying corticated branches, fungal stromata, phyllosphere habitats, and various soil ecosystems, attesting to their broad geographical distribution and adaptive capacity (Kredics et al., 2014; Jaklitsch and Voglmayr, 2015). *T. calcicola* aligns with the clade’s traits in its conidiophore branching, phialide arrangement, and green conidia. The newly described species, *T. xerophilum* and *T. exigua,* belong to the Harzianum clade, a cosmopolitan and widely distributed group. The clade displays a complex speciation history and diverse morphological characteristics (Atanasova et al., 2010; Druzhinina et al., 2010; Qin and Zhuang, 2017; Ye et al., 2023). Species within the Harzianum clade typically produce diverse pustules in culture, exhibiting variation in conidiophore morphology, phialide shapes, and conidial characteristics (Chaverri and Samuels, 2003; Jaklitsch, 2009; Zheng et al., 2021; Ye et al., 2023). Even in the present study, morphological characteristics of *T. xerophilum* and *T. exigua* also vary in the l/w ratio of phialides and arrangement. The taxonomy of the Harzianum clade was revised by Chaverri et al. (2015), who emphasized the need to use the secondary barcode *tef1-α* to accurately identify species within this complex. Subsequently, numerous species within this clade have been extensively reported, further enriching their diversity (Jaklitsch and Voglmayr, 2015; Qiao et al., 2018; Zhang and Zhuang, 2018; Phookamsak et al., 2019; Gu et al., 2020; Barrera et al., 2021; Cao et al., 2022, 2024; Ye et al., 2023).

*Trichoderma karsti* was robustly assigned to the Longibrachiatum clade, with high statistical support in phylogenetic analyses, and the species morphologically aligns with the diagnostic traits of the ‌Longibrachiatum‌ clade. In contrast to the other clades, the Longibrachiatum clade appears to be monophyletic (Samuels et al., 1998, 2012; Zhang and Zhuang, 2018). Samuels et al. conducted a comprehensive revision of this clade, describing eight new taxonomic units, including *Trichoderma aethiopicum*, and expanding the known species within the clade to 21, along with the development of a systematic identification key. Additionally, the re-description of species such as *T. parareesei* and the first identification of the sexual form of *T. gilliesii* significantly refined the taxonomic framework, laying a crucial foundation for future phylogenetic and functional studies. Following this methodological framework, an expanding array of species has been systematically identified and reported in this clade (Yabuki et al., 2013; Jaklitsch and Voglmayr, 2015; Qin and Zhuang, 2016; Zheng et al., 2021).

As a potential natural biocontrol resource or a contaminant of cultured mushrooms, *Trichoderma* has attracted considerable attention. Recent studies have documented *Trichoderma* diversity across multiple ecological niches, including: (1) plant-associated habitats (endophytic, epiphytic, and rhizosphere environments) (Xia et al., 2011; Mulatu et al., 2022); (2) fungal cultivation systems including edible mushroom substrates and medicinal fungi growth media (Wang et al., 2022; Cao et al., 2024); and (3) diverse ecosystems spanning alpine wetlands, forested areas, grasslands, wetlands, and agricultural landscapes (Tang et al., 2022; Dou et al., 2019). Sometimes, nationwide investigations of *Trichoderma* diversity were also conducted (Ahedo-Quero et al., 2024). Notably, *Trichoderma asperellum* appeared to be associated with the roots of the plant (Xia et al., 2011; Mulatu et al., 2022). Except for cultivation substrates of *Lentinula edodes* (Cao et al., 2024), *T*. *harzianum* was the predominant species in other natural ecosystems, either in agricultural or undisturbed soil. Its widespread distribution may be attributed not only to ecological plasticity but also to its competitive advantage in resource-poor environments, which may be a key factor in its success as a biocontrol agent. Previous studies have also demonstrated that both *T. harzianum* and *T. asperellum* can promote seed germination, highlighting their practical potential in agriculture (Muradov et al., 2025).

In our survey, the most abundant species was also *T*. *harzianum* with an isolation frequency of 26.31%, which is close to 23% in alpine wetlands with a similar arid and barren environment to karst desert soil. This consistency suggests that *T. harzianum* may exhibit habitat-specific adaptation to stressful environments. Future comparative studies across different ecosystems may further elucidate its ecological preferences and functional potential. Recent studies have shown that *Trichoderma* spp. significantly enhance organic matter decomposition by increasing CO₂ release and residue turnover (Organo et al., 2022), suggesting that they may play an important role in nutrient cycling and ecosystem recovery in karst desertification soils.

Nevertheless, this study has some limitations. The culture-dependent approach used here may underestimate total fungal diversity by missing unculturable or slow-growing taxa. In addition, all samples were collected from a soil depth of 5–10 cm, potentially overlooking fungi present in deeper horizons or at the rhizoplane. Future studies should incorporate high-throughput sequencing and functional assays to comprehensively characterize the ecological roles and adaptive mechanisms of *Trichoderma* in karst desert environments.

## Data Availability

The original contributions presented in the study are publicly available. This data can be found here: https://www.ncbi.nlm.nih.gov/, accession numbers PV344624, PV702217, PV344618, and PV344620.
